# Does Circular Stapler Size in Surgical Management of Esophageal Cancer Affect Anastomotic Leak Rate? 4-Year Experience of a European High-Volume Center

**DOI:** 10.3390/cancers12113474

**Published:** 2020-11-22

**Authors:** Dolores T. Müller, Benjamin Babic, Veronika Herbst, Florian Gebauer, Hans Schlößer, Lars Schiffmann, Seung-Hun Chon, Wolfgang Schröder, Christiane J. Bruns, Hans F Fuchs

**Affiliations:** Department of General, Visceral, Cancer and Transplant Surgery, University of Cologne, Kerpener Str. 62, D-50937 Cologne, Germany; dolores.mueller@uk-koeln.de (D.T.M.); benjamin.babic@uk-koeln.de (B.B.); veronika.herbst3@gmail.com (V.H.); florian.gebauer@uk-koeln.de (F.G.); hans.schloesser@uk-koeln.de (H.S.); lars.schiffmann@uk-koeln.de (L.S.); seung-hun.chon@uk-koeln.de (S.-H.C.); wolfgang.schroeder@uk-koeln.de (W.S.); christiane.bruns@uk-koeln.de (C.J.B.)

**Keywords:** esophagectomy, esophageal anastomosis, minimally invasive surgery

## Abstract

**Simple Summary:**

One of the most severe postoperative complications after a transthoracic esophagectomy for esophageal cancer is a leakage of the anastomosis created between the remnant esophagus and the stomach. There is substantial debate on which surgical technique and which stapler are the best. The aim of this study was to retrospectively analyze whether the stapler diameter had an impact on postoperative anastomotic leak rates during a 4-year time frame from 2016 to 2020. A total of 632 patients (open, hybrid, and totally minimally invasive esophagectomy) met the inclusion criteria. A total of 214 patients underwent an anastomosis with a 25 mm stapler vs. 418 patients with a 28 mm stapler. Anastomotic leak rates were 15.4% vs. 10.8%, respectively. Stapler size should be chosen according to the individual anatomical situation of the patient and may be of higher relevance in patients undergoing totally minimally invasive reconstruction.

**Abstract:**

Anastomotic leak is one of the most severe postoperative complications and is therefore considered a benchmark for the quality of surgery for esophageal cancer. There is substantial debate on which anastomotic technique is the best for patients undergoing Ivor Lewis esophagectomy. Our standardized technique is a circular stapled anastomosis with either a 25 or 28 mm anvil. The aim of this study was to retrospectively analyze whether the stapler diameter had an impact on postoperative anastomotic leak rates during a 4-year time frame from 2016 to 2020. A total of 632 patients (open, hybrid, and totally minimally invasive esophagectomy) met the inclusion criteria. A total of 214 patients underwent an anastomosis with a 25 mm stapler vs. 418 patients with a 28 mm stapler. Anastomotic leak rates were 15.4% vs. 10.8%, respectively (*p* = 0.0925). Stapler size should be chosen according to the individual anatomical situation of the patient. Stapler size may be of higher relevance in patients undergoing totally minimally invasive reconstruction.

## 1. Introduction

Due to its increasing incidence, a curative treatment of esophageal carcinoma has gained more importance than ever in recent years. For locally advanced but resectable carcinomas, a transthoracic esophagectomy with reconstruction using a gastric conduit and a high intrathoracic anastomosis (Ivor Lewis esophagectomy) depicts the current curative treatment of choice, mostly in a multimodal setting [1]. Despite improvements of perioperative care and surgical technique, this surgical procedure is still related to specific risks, such as anastomotic leak, conduit necrosis, chylothorax, and recurrent nerve injury. In particular, anastomotic leak, as one of the most severe and early postoperative complications, is considered a benchmark for the quality of the esophagectomy and is known to increase postoperative mortality and morbidity, leading to a decreased long-term survival [2,3,4]. Many surgical factors, including procedure type, localization of the anastomosis, and operative technique, are known to affect the integrity and quality of the anastomosis, and there is substantial debate on which anastomotic technique is the best for patients undergoing Ivor Lewis esophagectomy [5,6]. Our standardized technique and the most common technique in minimally invasive surgery is a circular stapled end-to-side anastomosis with purse string using either a 25 or 28 mm anvil. The current literature has shown this technique to be safe and efficient, leading to a comparatively low anastomotic leak rate of 14% in the EsoBench database [4]. The aim of this study was to retrospectively analyze whether the stapler diameter had an impact on postoperative anastomotic leak rates according to Esophagectomy Complications Consensus Group (ECCG) criteria during a 4-year time frame from 2016 to 2020 at our certified center of excellence for surgery of upper gastrointestinal cancer. 

## 2. Results

A total of 632 patients met the inclusion criteria. In 214 patients (34%), a 25 mm circular stapler was used for the construction of a transthoracic esophagogastric anastomosis, and in 418 patients (66%), a size of 28 mm. For further analysis of results, the patients were grouped according to stapler size (25 mm—small; 28 mm—large). Demographic and oncological data of both patient cohorts are shown in Table 1. In addition, a statistical comparison of the baseline characteristics of both groups was performed, and the *p*-values are shown. 

**Figure 1:**
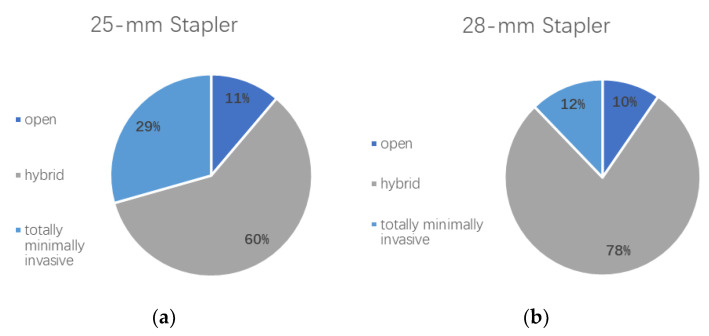
(**a**) Distribution of surgical approach in the 25 mm stapler patient group. Percentages of patients undergoing an Ivor Lewis esophagectomy using an open, hybrid, or totally minimally invasive approach are shown. (**b**) Distribution of surgical approach in the 28 mm stapler patient group. Percentages of patients undergoing an Ivor Lewis esophagectomy using an open, hybrid, or totally minimally invasive approach are shown.

Furthermore, the operative approach was analyzed for both groups and compared with each other. Figure 1 shows the distribution of open, hybrid, and totally minimally invasive procedures among patient cohorts.

While an open approach was used equally often in both cohorts (*p* = 0.6809), a hybrid approach was more often performed in the 28 mm group (*p* < 0.0001), compared with a totally minimally invasive approach, which was more frequently performed in the 25 mm group (*p* < 0.0001). In line with these findings, a two-stage procedure was more often used in the 28 mm stapler size group (6.5% vs. 11.5%; *p* = 0.0490). 

A total of 72 patients in the given time frame were operated on using an either completely robotic or hybrid robotic approach. Thirty-one (14.5%) of the patients in the 25 mm stapler group were operated on using a robotic technique compared with 41 (9.8%) in the 28 mm stapler group. No statistically significant difference was shown for the utilization of a robotic technique between both groups (*p* = 0.0863). 

A total of 114 patients in the given time frame were operated on using a totally minimally invasive approach (laparoscopic or robotic gastrolysis/thoracoscopic or robotic esophagectomy). Sixty-three (29.4%) of the patients in the 25 mm stapler group were operated on using a totally minimally invasive technique, compared with 51 (12.2%) in the 28 mm stapler group. A totally minimally invasive technique was significantly more often used in the smaller stapler group (*p* < 0.0001). 

### Postoperative Complications 

Table 2 shows the severity of postoperative complications classified according to Clavien–Dindo (CD) of our patient groups. In addition, *p*-values were obtained to analyze whether statistically significant differences between postoperative outcomes of both cohorts were present.

The median length of stay (LOS) was 15 days in both groups with a range of 9–112 days (standard deviation (SD) 12) in the small stapler group and a range of 7–99 days (SD 11) in the large stapler group with no statistically significant difference between the groups (*p* = 0.3993). 

**Figure 2:**
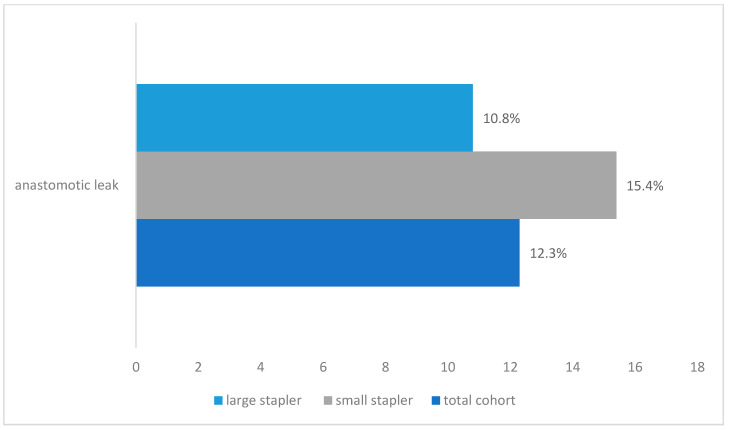
Anastomotic leak rates for the small stapler size (25 mm), the large stapler size (28 mm), and the overall cohort shown as percentages.

An anastomotic leak was detected in a total of 78 patients (12.3%), 33 in the small stapler group and 45 in the large stapler group. Figure 2 depicts the anastomotic leak rates among patient cohorts. No statistical significance was noted between the groups (*p* = 0.09878); however, a trend approaching statistical significance shows that anastomotic leaks were more frequent in the small stapler size group.

Further details on demographic information, preoperative comorbidities, and risk factors of patients who developed an anastomotic leak are shown in Table 3. 

In addition, independent predictors of anastomotic leak were identified by a multivariate logistic regression analysis adjusting for stapler size, operative technique, gender, type of cancer, neoadjuvant therapy, tobacco, alcohol consumption, BMI, and cardiac comorbidities. Besides operative approach (open, hybrid, totally minimally invasive) and gender, no other independent predictors for anastomotic leak were found (*p* > 0.05). With totally minimally invasive approach set as a reference, both other approaches had a significantly higher leak rate when adjusted for all factors mentioned above (*p* < 0.05).

Table 4 shows further details about the distribution of the type of anastomotic leaks as well as the severity of complications using the Clavien–Dindo classification among patients that developed an anastomotic leak. The anastomotic leak rate for a Type II leak was 11.2% in the 25 mm group vs. 8.4% in the 28 mm group and 4.2% vs. 2.2% for Type III leaks, respectively. In addition, the percentage of patients with a certain CD class from the total of anastomotic leaks among the respective stapler sizes is shown. No patient with an anastomotic leak was classified as CD I or II. To investigate how many patients with an anastomotic leak developed organ failure, we included a subgroup analysis of patients classified as CD ≥ IV. 

## 3. Discussion

Anastomotic leakage is among the most feared complications in surgery due to its consequences, especially in esophageal cancer surgery. Many technical variations in performing the esophagogastric anastomosis are still used without expert consensus. Our institution has contributed significantly in the past to find innovative and new ways using minimally invasive technology to treat these complications with an interventional approach and mostly without redo surgery, leading to more ECCG Type II anastomotic leaks in recent years [7,8]. Schröder et al. have published in their recent multicenter analysis of high-volume centers from 2011 to 2016 a leakage rate of 13.9% for an intrathoracic circular stapled anastomosis. Our own data from 2016 to 2020 in this present study show an overall leakage rate of 12.3%, meeting the benchmarks in Schröder’s and Schmidt’s studies [2,4]. When looking into more detail as presented above, we were able to show that in addition to the technology and technique used, even the stapler size may play an essential role in developing an anastomotic leak. In this context, it is important to note that anatomical reasons may play an essential role when choosing the stapler size, and sometimes both options are technically not possible. 

Interestingly, we were able to show that a 28 mm stapler was overall significantly more often used at our institution. This result may be biased by the fact that more hybrid than totally minimally invasive procedures were performed in this collective, as the 25 mm stapler was the more commonly chosen technology in the totally minimally invasive subgroup. Also, patients with squamous cell carcinoma more often underwent a 25 mm anastomosis, a fact that can be attributed to the usually higher mediastinal location of the tumors and anatomical reasons not to perform a 28 mm stapled anastomosis.

Few previous studies have focused on the technical factor of the stapler diameter itself, but more often evaluated general technical options, such as circular, linear, and handsewn (technical factors), and anatomical options, such as intrathoracic vs. cervical (anatomical factors) [4,5]. Whereas Markar et al. published a meta-analysis in 2013 showing no significant differences among the technical factors, they were able to show significant differences and an almost fivefold increased leakage rate for cervical vs. intrathoracic anastomosis. In contrast, Schröder’s analysis of the EsoBenchmark database showed no difference among the anatomical factors. Even if the difference in leakage rate in our patient collective (10.8% vs. 15.4%) is in favor of the 28 mm stapler group, this technical factor was only nearing statistical significance (*p* = 0.0925).

In our analysis of complications, focusing on the patients with an anastomotic leak (Table 4), we looked at the severity of the leaks according to the ECCG group [9]. No significant difference was found between the two analyzed stapler groups. Nevertheless, there was again a trend of less severe leaks in favor of the 28 mm staplers. No clear differences could be shown for the Clavien–Dindo score between the groups. 

In our study, we found a relatively high number of CD ≥ IIIa complications, namely, 51.4% (28 mm) and 55.6% (25 mm). This exceeds the benchmarks set by Schmidt et al. with 30.8%, defined as the “best possible outcome” [2]. Truly, our collective does not comprise a selection of patients with low comorbidities, and esophagectomy was performed both by experts and by trainees under expert supervision at our institution, meaning that our results represent an unbiased, unselected analysis of a prospective cohort. In addition, complications at our institution are thoroughly recorded according to ECCG guidelines, meaning that postoperative interventions such as chest tube placement or postoperative EGD are automatically classified as a IIIa complication. As postoperative endoscopic interventions are considered a “standard of care” in some other centers, these might not be classified in the same way everywhere.

## 4. Materials and Methods 

### 4.1. Patients 

Our academic center is a certified center of excellence for surgery of the upper gastrointestinal tract with more than 250 upper gastrointestinal cancer surgeries being performed annually. All patients undergoing esophagectomy for esophageal cancer in our high-volume center are entered into an IRB-approved prospective database. A retrospective chart review was performed for all patients undergoing an Ivor Lewis esophagectomy for esophageal cancer from May 2016 to May 2020. Patients were included in the analysis if a 25 or 28 mm circular stapler was used for the esophagogastric transthoracic anastomosis. An intraoperative subjective assessment of the patient’s anatomy was used to choose the appropriate stapler size. Patients with handsewn anastomoses or other stapler diameters were excluded from the analysis. Retrospective analysis of our prospectively collected data was conducted with approval from the ethical committee at the University of Cologne (IRB reference 13-091). Demographics, endoscopic findings, and biopsies at different follow-up time points, as well as tumor histology and stage, were recorded in our prospective database. 

### 4.2. Assessment of Postoperative Complications

The Clavien–Dindo classification was used to classify the severity of postoperative complications [10]. In addition, our institution contributes to the well-established database of the Esophagectomy Complications Consensus Group (ECCG), which provides a standardized and international assessment of complications following esophagectomy [9]. Therefore, definitions established by the ECCG are used at our clinic to ensure precise documentation. An anastomotic leak was defined as a “full thickness GI defect involving esophagus, anastomosis, staple line, or conduit irrespective of presentation or method of identification.” Further subgrouping into three types was applied with Type I being a local defect requiring no change in therapy or being treated medically or with dietary modification, Type II being a localized defect requiring interventional but not surgical therapy, and Type III being a localized defect requiring surgical therapy. Length of hospital stay was calculated in days from the day of the surgical procedure to discharge of the patient. 

### 4.3. Treatment Pathway of Patients with Resectable Esophageal Cancer 

Treatment of patients with esophageal cancer at our National Center of Excellence follows a standardized protocol in line with national and international guidelines [1,11,12,13]. Following restaging, usually 4–6 weeks after neoadjuvant therapy, either a standardized Ivor Lewis esophagectomy with reconstruction using a gastric conduit and a high thoracic esophagogastric anastomosis is performed at our institution, or if suitable, patients with an adenocarcinoma of the gastroesophageal junction Siewert Type II are enrolled into the CARDIA trial, which aims to compare the oncological and surgical outcome after transthoracic esophagectomy and transhiatal extended gastrectomy [14,15]. For a transthoracic esophagectomy, a hybrid procedure (abdominal part—laparoscopically/thoracic part—open) depicts the current standard at our institution. Whenever possible, however, dependent on the patient’s anatomy and whether the patient is classified as low risk, a totally minimally invasive approach is chosen (abdominal part—laparoscopically/thoracic part—thoracoscopically). Our thorough risk assessment preoperatively includes a standardized and validated risk scoring system [16]. In addition, the DaVinci Xi robotic surgical system (Intuitive Surgical, Inc., Sunnyvale, CA, USA) is available at our clinic since February 2017. A robotic approach is often especially used for the thoracic part, as the great advantage of the system becomes evident during the thoracic dissection [17]. Furthermore, complete robotic and minimally invasive Ivor Lewis esophagectomies are increasingly performed at our institution. 

### 4.4. Surgical Technique—Abdominal Part

The following steps for preparation of the gastric conduit are performed in a standardized fashion using either a robotic or a laparoscopic approach. Our standardized steps of the operation are the same for the robotic and the laparoscopic procedure: The patient is placed in a French and anti-Trendelenburg position. For the robotic approach, an 8 mm DaVinci trocar is inserted through a supraumbilical median incision using the open technique, and a pneumoperitoneum is established. Four additional trocars are then placed, one 5 mm trocar on the right and one 12 mm trocar on the left edge of the costal arch, a 12 mm trocar in the right upper abdomen and an 8 mm trocar in the left upper abdomen depicting the standard for a minimally invasive robotic DaVinci gastrolysis. If performed laparoscopically, one 5 mm and four 11 mm abdominal ports are used. A 45-degree angled scope (5 mm Stryker indocyanine green (ICG) or robotic 8 mm Intuitive ICG) is inserted through the subxiphoidal trocar. The hiatus is then exposed by elevating the liver with a Cuschieri retractor through the right 5 mm trocar. From here, the peritoneum on the right diaphragmatic crus is incised, and the lower mediastinum outside the hernia sac is dissected and circumferentially mobilized up to the left diaphragmatic crus to dissect the lower esophagus. Opening the right and the left pleura is avoided at any time during the hiatal dissection. Dissection of the lymph nodes along the lesser curvature of the stomach onto the stomach wall follows. The upper margin of the retroperitoneal pancreas is now exposed and can be inspected. A D2 lymphadenectomy following the hepatic ligament, the common hepatic artery, along the celiac trunk continuing along the splenic artery and of the retroperitoneum is performed. The left gastric artery and the left gastric vein are ligated, clipped, and divided. The right gastric artery is preserved. Subsequently, lymph nodes along the retroperitoneum via the crus of the diaphragm up to the lower mediastinum are mobilized, and the lymphadenectomy is completed above the splenic artery all the way up to the hilum of the spleen. Opening the gastrocolic omentum access to the omental bursa is gained, and the greater curvature of the stomach is mobilized starting from the corpus region beyond the epiploic vessels toward the left crus of the diaphragm, while the gastroepiploic arcade is preserved, and the short gastric vessels are divided until visualization of the left diaphragmatic crus is achieved. To later create an omentum wrap covering the anastomosis, a part of the greater omentum just below the spleen is preserved. Mobilization is completed by separating the colon all the way until the splenic flexure, confirming sufficient blood supply for the greater curvature. Dissection at the gastric crow’s foot region is performed followed by the construction of the gastric conduit. A tristapler (Endo Gia (Covidien), violet, 45 mm) is applied for the first bite of the construction of the gastric sleeve. Using at least two additional Endo Gia 60 mm violet stapling magazines, construction of the gastric conduit is completed. Intraoperative angiography using indocyanine green (ICG) can, in combination with the robotic DaVinci Xi system of the laparoscopic Stryker system, demonstrate sufficient blood supply of the fundus by showing the gastroepiploic vessels via fluorescence.

### 4.5. Surgical Technique—Thoracic Part 

The following steps for completion of the esophagectomy and reconstruction of the gastrointestinal passage using a gastric conduit are performed in a standardized fashion using either a minimally invasive thoracoscopic, robotic, or open approach: The patient is placed in a left lateral semiprone position for a robotic procedure or in a left lateral decubitus position for an open procedure. Using a double-lumen intubation, artificial atelectasis of the right lung is achieved. Our standardized steps of the operation are the same for the robotic or the open procedure. For a robotic approach, three DaVinci ports and two assistance ports are placed on the right according to the standard, and the robot is docked from the patient’s right side, creating a view from the left for the operating surgeon. A right-sided transthoracic approach is used for an open procedure. Using the robotic monopolar cautery hook, the pulmonary ligament is dissected with the lymph nodes adhering to the esophagus upward toward the pericardial layer and the azygos vein. Using a tristapler (Endo Gia (Covidien), gold, 45 mm), the azygos arch is divided. The thoracic duct is identified and clipped with two polymer clips (Grena Click’aV^®^). Dissection of the periesophageal fat tissue along the aorta dividing small aortic branches and along the pericardium is performed. Especially when using the robotic technique, a radical but controlled dissection of the carinal, retrotracheal, and paratracheal tissue can be performed. Vagal and recurrent nerves are preserved during this step. Opening the hiatus, a connection to the abdominal surgical field is made. A monofilament purse string suture is performed, and the gastric conduit is pulled into the right thoracic cavity. If a minimally invasive approach is chosen, a minithoracotomy of 7 cm length is then created from the incision of the 12 mm upper assistance trocar, and an Alexis S wound protector/retractor (Alexis Laparoscopic System, Applied Medical) is inserted. 

### 4.6. Surgical Technique—The Esophagogastric Anastomosis 

**Figure 3:**
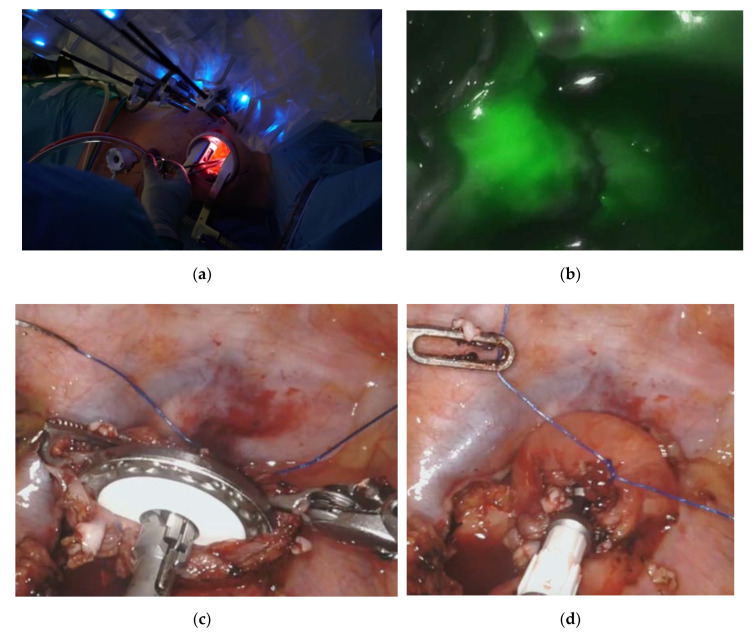
Robotic-assisted minimally invasive esophagectomy. The top left picture (**a**) shows a minithoracotomy of 7 cm length created from the incision of the 12 mm upper assistance trocar and secured with an Alexis S wound protector/retractor. The top right picture (**b**) shows intraoperative angiography using indocyanine green (ICG). The bottom pictures display how the prepared purse string suture is used to suture the stapler head into the esophageal remnant (**c**,**d**).

Either a 25 or 28 mm stapler head depending on the patient’s anatomy is inserted and guided into the esophagus. The prepared purse string suture is used to suture the stapler head into the esophageal remnant. If necessary, a second purse string suture may be placed. Figure 3 shows the setup for the creation of the esophagogastric anastomosis. The gastric conduit is then gently pulled upward into the chest. We always ensure that the fundus lies alongside with the esophageal stump without tension, which further proofs a sufficient length of the conduit. Intraoperative angiography using ICG can be again used with the robotic system to demonstrate sufficient blood supply of the graft. If a robotic approach is chosen, the DaVinci is then disconnected, and the assistant surgeon holds the camera similar to a thoracoscopic approach. Using a variable number of loads of the Endo GIA stapler (Covidien), preparation of the gastric conduit is completed. The specimen is removed and preserved for histopathologic evaluation. A 25 or 28 mm stapler is inserted through the minor curvature of the stomach, and an esophagogastric anastomosis is made, retrieving two complete donuts. Another Endo GIA stapler load is used to staple off the open end of the stomach. The previously prepared omentum wrap is then used to cover the anastomosis. In addition, final control of blood perfusion using ICG fluorescence can be used. 

### 4.7. Data Analysis and Statistical Evaluation 

For analysis of data, patients were divided into two groups based on CS (circular stapler) size (“small” = 25 mm circular stapler and “large” = 28 mm circular stapler). In addition, a subgroup analysis of patients who underwent a totally minimally invasive and a robotic esophagectomy was performed. Continuous variables are presented as means and range. Categorical data are presented as numbers and percentages. Student’s *t*-test (for continuous variables) and Fisher’s exact test (for nominal or categorical variables) were used for all bivariate analyses. Independent predictors of anastomotic leak were identified by a multivariate logistic regression analysis. All tests were two-sided, with statistical significance set at *p* ≤ 0.05. Data were analyzed by GraphPad Software (San Diego, CA, USA) and SPSS Statistics for Mac (version 21, SPSS).

## 5. Conclusions

This large single-center analysis clearly defines anastomotic leak rates of a standardized, unselected consecutive patient cohort in a high-volume center. We highly recommend that stapler size be always chosen according to the individual anatomical situation of the patient, but when in doubt, we suggest choosing the larger diameter. This suggestion may be of even higher relevance to patients undergoing minimally invasive thoracic reconstruction.

## Figures and Tables

**Table 1:** Demographic characteristics and oncological data of the patients undergoing an Ivor Lewis esophagectomy for esophageal cancer with either a 25 or 28 mm circular stapler. The *p*-values for statistical comparison of the baseline characteristics of both groups were calculated.

	25 mm		28 mm		
	Total/Mean	(%)/Range	Total/Mean	(%)/Range	*p*-Value
**Patients**	214	34	418	66	<0.0001
**Male/female**	150/64	(70.1)/(29.9)	377/41	(90.2)/(9.8)	<0.0001
**Age (years)**	63	29–91	63	34–85	0.8431
**BMI (kg/m^2^)**	25.68	15–46.71	26.87	14.13–48.44	0.0037
**Pathology**					
**Adenocarcinoma**	157	(73.4)	345	(82.5)	0.0092
**Squamous cell carcinoma **	57	(26.6)	70	(16.8)	0.0045
**Other**	0	(0)	3	(0.7)	0.5546
**Neoadjuvant Chemotherapy**					
**None**	32	(15)	54	(12.9)	0.54
**CROSS**	137	(64)	240	(57.4)	0.1231
**FLOT **	40	(18.7)	113	(27.1)	0.0238
**Other**	5	(2.3)	11	(2.6)	1

BMI: body mass index; CROSS and FLOT are well defined neoadjuvant treatments.

**Table 2:** Severity of postoperative complications among patient cohorts. The Clavien–Dindo classification was used to objectify the severity of postoperative complications among both patient cohorts. *p*-Values were calculated to analyze whether statistically significant difference between both stapler sizes was present. In addition, further analysis of patients with severe postoperative complications, here classified as CD ≥ IIIa, was performed.

	25 mm Stapler		28 mm Stapler		
	*n*	(%)	*n*	(%)	*p*-Value
**CD 0**	66	(30.8)	151	(36.1)	0.2152
**CD I**	10	(4.7)	21	(5.1)	1
**CD II**	19	(8.9)	31	(7.4)	0.5354
**CD IIIa**	79	(36.9)	140	(33.5)	0.4268
**CD IIIb**	15	(7.1)	26	(6.2)	0.7340
**CD IVa**	14	(6.5)	24	(5.7)	0.7248
**CD IVb**	8	(3.7)	13	(3.1)	0.6474
**CD V**	3	(1.4)	12	(2.9)	0.4075
**CD ≥ IIIa**	119	(55.6)	215	(51.4)	0.3545

**Table 3:** Demographic information, comorbidities, and risk factors of patients who developed an anastomotic leak. Data are shown for both subgroups (25 mm and 28 mm circular staplers), and percentages of patients from the respective subgroups were calculated.

	Anastomotic Leak				
	25 mm Stapler Size		28 mm Stapler Size		
	Total	(%)	Total	(%)	*p*-Value
**Patients**	33	(100)	45	(100)	-
**Obesity** **(BMI > 30 kg/m^2^)**	6	(18.2)	8	(17.8)	1
**Tobacco**					
**Ex-smoker**	17	(51.5)	15	(33.3)	0.1617
**Smoker**	7	(21.2)	12	(26.7)	0.6068
Alcohol consumption *					
**None**	18	(54.5)	27	(60)	0.6502
**1–3 ×/week**	10	(30.3)	10	(22.2)	0.4432
**Daily**	5	(15.2)	5	(11.1)	0.7351
**Cardiac comorbidities**					
**Coronary artery disease**	8	(24.2)	7	(21.2)	0.3911
**Arterial hypertension**	22	(66.7)	33	(73.3)	0.6176
**Atrial fibrillation**	4	(12.1)	5	(11.1)	1
**Pulmonary comorbidities**					
**COPD**	2	(6.1)	3	(6.7)	1
**FEV1 < 80%**	5	(15.2)	11	(24.4)	0.4004
**VCmax < 80%**	6	(18.2)	9	(20)	1
**Other comorbidities**					
**Liver disease**	3	(9.1)	3	(6.7)	0.6937
**Renal failure** **(GFR < 60 mL/min)**	3	(9.1)	4	(8.9)	1
**Diabetes**	4	(12.1)	6	(13.3)	1

* Information was given voluntarily; therefore, not all patients answered this question. COPD: chronic obstructive pulmonary disease, FEV1: forced expiratory pressure.

**Table 4:** Anastomotic leak types and severity of postoperative complications among patients that developed an anastomotic leak. Stapler sizes of 25 and 28 mm were analyzed separately, and *p*-values for statistical comparison of both groups were calculated. Percentages were calculated as percentage from the cohort that developed an anastomotic leak.

	25 mm Stapler		28 mm Stapler		
	*n*/Median	(%)/Range	*n*/Median	(%)/Range	*p*-Value
**Total**	33	(100)	45	(100)	-
**Type I**	0	(0)	1	(2.2)	1
**Type II**	24	(72.7)	35	(77.8)	0.79
**Type III**	9	(27.3)	9	(20)	0.5876
**Clavien–Dindo Classification**					
**CD IIIa**	14	(42.4)	21	(46.7)	0.8188
**CD IIIb**	4	(12.1)	6	(13.3)	1
**CD IVa**	9	(27.3)	9	(20)	0.5876
**CD IVb**	3	(9.1)	6	(13.3)	0.7259
**CD V**	3	(9.1)	3	(6.7)	0.6937
**CD ≥ IV**	15	(45.5)	18	(40)	0.6502
**Length of Stay**					
**LOS**	36	16–112	30	13–99	0.3118
